# HIV risk and psychological distress among female entertainment workers in Cambodia: a cross-sectional study

**DOI:** 10.1186/s12889-016-2814-6

**Published:** 2016-02-09

**Authors:** Carinne Brody, Pheak Chhoun, Sovannary Tuot, Khuondyla Pal, Kolab Chhim, Siyan Yi

**Affiliations:** 1 Center for Global Health Research, Public Health Program, Touro University California, 1310 Club Drive, Vallejo, CA 94592 USA; 2 Center for Population Health Research, KHANA, No. 33, Street 71, Tonle Bassac, Chamkar Morn, P.O. Box 2311-PP3, Phnom Penh, Cambodia

**Keywords:** Female entertainment workers, Sex work, Psychological distress, Mental health, HIV, Cambodia

## Abstract

**Background:**

In Cambodia, there has been an increase in entertainment work as a result of the breakdown of the traditional brothel-based sex industry, presenting new challenges to addressing the health issues and needs of people working in the entertainment industry. This study aims to identify factors associated with psychological distress among female entertainment workers (FEWs) in Cambodia.

**Methods:**

A two-stage cluster sampling method was used to randomly select 657 FEWs from entertainment establishments in Phnom Penh and Siem Reap in April and May 2014 for interviews using a structured questionnaire. Psychological distress was measured using the General Health Questionnaire (GHQ-12), and multivariate logistic regression analysis was conducted.

**Results:**

Almost half of FEWs (43.2 %) had a higher level of psychological distress (GHQ-12 > 3), while 19.5 % reported having suicidal thoughts, and 7.3 % reported having attempted to commit suicide in the past 3 months. Controlling for confounding factors, women with a higher level of psychological distress were significantly more likely to rate their overall health (AOR = 1.88, 95 % CI 1.20 to 2.94) and quality of life (AOR = 2.39, 95 % CI 1.47 to 3.87) as poor. They were also significantly more likely to have suicidal ideation (AOR = 2.41, 95 % CI 1.45 to 3.76), rate their HIV risk as higher than the general population (AOR = 0.48, 95 % CI 0.31 to 0.74), have been forced to drink at work (AOR = 1.77, 95 % CI 1.19 to 2.62), have had clients requesting not to use a condom (AOR = 3.48, 95 % CI 1.14 to 10.62), be not able to find condoms when they needed it (AOR = 0.64, 95 % CI 0.45 to 0.93), have had a family member who said hurtful things to them during childhood (AOR = 1.84, 95 % CI 1.24 to 2.75), and have had a parent or guardian who had been physically abused (AOR = 1.93, 95 % CI 1.34 to 2.82).

**Conclusions:**

FEWs in Cambodia experience high levels of psychological distress, which likely stems from both past negative experiences and current working conditions. For women that are experiencing psychological distress, intervention programs aimed at improving mental health should specifically address substance use, condom availability and negotiation skills, and suicide risk.

## Background

Female entertainment workers (FEWs) are typically young women in developing countries, particularly in Asia, who work at establishments such as karaoke bars, restaurants, beer gardens or massage parlors. Entertainment work is often associated with sex work [1, 2], where women employed as singers, beer promoters, or masseuses sell sex to male patrons to supplement their income. FEWs, in particular those who participate in sex work, are at greater risk for health problems than the general women population due to occupational hazards such as engaging in unsafe sex, exposure to violence, and drug use [1–3]. This group can be difficult to reach with health services due to the hidden and stigmatizing nature of their work [2, 3].

Despite these occupational stressors, mental health of female sex workers (FSWs) is understudied [4]. The most relevant data on the mental health of this population come from studies assessing the mental health of FSWs. Available evidence suggests that FSWs experience worse mental health outcomes than the general public such as high rates of anxiety, depression, posttraumatic stress disorder (PTSD), and psychological distress [4–7]. These poor mental health outcomes have been associated with experiences of violence, childhood trauma, drug addiction, and the experience of stigma as reported in a review of the literature [8]. The existing evidence does not establish a temporal relationship but suggests that there is likely a bi-directional relationship between poor mental health and sex work.

Studies suggest that poor mental health among FSWs can lead to more health risk behaviors such as substance use or unsafe sex. In China and Iran, studies have shown that FSWs with severe depressive symptoms were less likely to use condoms consistently [9, 10]; and in the United States and Puerto Rico, FSWs exhibiting depressive symptoms were more likely to engage in unsafe drug use practices such as needle sharing [11–13]. These studies suggest that FSWs experiencing poor mental health are at greater risk of contracting HIV.

In Cambodia, the patterns of entertainment work have changed dramatically, which complicates the provision of any type of health services. The global financial crisis of 2007–08, which lead to high unemployment in the garment sector, and the 2008 ‘Law on Suppression of Human Trafficking and Sexual Exploitation’ that banned brothel-based sex work [14] have created a large influx of young women working in the entertainment and informal sex industry. Today, more young women than ever are working in karaoke parlors, restaurants, beer gardens, massage parlors, bars, or nightclubs. At these establishments, some women engage in sexual relationships in exchange for money, gifts, or other benefits for themselves or their family [15].

Many FEWs in Cambodia come from low-income rural households, have low education levels, and have received only limited health education [14]. As urban migrants, these women face a changing social context and increased stigma with less social support [16, 17]. These factors may result in an increasing vulnerability to mental health problems for this population. The breakdown of the traditional brothel-based sex industry and the emergence of indirect methods of selling sex through entertainment venues have created challenges to identifying FEWs and providing services to address their health issues and needs, including sexual and reproductive and mental health.

The nature of FEWs is unique to the Cambodian context, and their health situations and behaviors are somewhat different from and more complex than the situations of FSWs in most other countries. In addition, supportive services for mental health for the general public in Cambodia are extremely limited [18]. Reaching FEWs with any type of health services can be difficult, and with the limited available mental health services in the country, addressing the mental health needs of this population is very challenging. Identifying FEWs at higher risk for psychological distress based on risk factors may be one way to maximize scarce resources and provide comprehensive mental and physical health services to the most vulnerable women. This study aims to identify the factors associated with psychological distress among FEWs in Cambodia.

## Methods

### Study sites, sampling, and training

Data used for this study were collected in April and May 2014 as part of an impact evaluation of the Sustainable Action against HIV and AIDS in Communities (SAHACOM) Project implemented by KHANA, the largest national non-governmental organization providing integrated HIV prevention, care, and support services at the community level in Cambodia. Participants were recruited from entertainment venues and hotspots from a list obtained from KHANA’s partners who implemented the SAHACOM programs in the capital city of Phnom Penh and Siem Reap province. The number of FEWs in Phnom Penh and Siem Reap represents approximately 70 % of the total FEWs population in Cambodia [19]. The sample size was proportionally allocated to the number of FEWs in two study sites. The details of the study have been published elsewhere [20, 21].

A two-stage cluster sampling method was used to select participants from the two provinces. At the first stage, the probability proportional-to-size sampling method was used to select entertainment venues and hotspots from the list. At the second stage, a proportionate number of FEWs were randomly selected from the each selected venue and hotspot. Inclusion criteria for the study included: (1) biologically female; (2) at least 18 years of age; (3) able to present themselves on the day of the interview; and (4) able to provide consent to participate in the study.

All research team members were trained for three days on the study methods, interview techniques, privacy assurance, and confidentiality. The training also addressed quality control strategies, such as rechecking and reviewing the questionnaires after administration and resolving issues that might arise during the fieldwork. Data collection team leaders were encouraged to perform regular reviews on the progress of the field work and communicate any issues that may occur during the data collection.

### Questionnaire development and measurements

Cambodian research staff members that regularly work with FEWs developed a structured questionnaire in English and then translated into Khmer, the national language of Cambodia. The Khmer questionnaire was back-translated and pretested with a sample of 10 FEWs in Phnom Penh to ensure that the wording and contents were culturally suitable and clearly understandable for the respondents. We also sought for comments from experts working on HIV in key populations in Cambodia, and the questionnaire was finalized based on their feedback and findings from the pilot study.

Standardized tools were adapted from previous studies in the same population [22], the most recent Cambodia Demographic and Health Survey [23], as well as from other studies in Cambodia [24, 25]. Socio-demographic characteristics included age, marital status, completed years of formal education, average monthly income, living situations, types of establishment they were working for, and duration they had worked in the current career as well as in the current venue. In addition, we also collected information on self-perception of the level of HIV risk compared to the general population, self-rated overall health and quality of life, suicidal thoughts, and suicidal attempts in the past 3 months.

To measure substance use, participants were questioned about whether they used any kinds of alcohol and illicit drugs in the past 3 months. They were also asked to report the average number of days they got drunk and types of illicit drugs they used in the past month.

Several variables on sexual and reproductive health were measured including number of sexual partners in the past 12 months, number of partners with whom they had sexual intercourse in exchange for money or gifts (commercial partners), number of partners with whom they had sexual intercourse not in exchange for money or gift (non-commercial partners), and condom use with both types of sexual partners in the past 3 months. For condom use, we asked, “During the past 3 months, how often have you used condoms when you had sex with your sweetheart (regular non-commercial partner)?” The same question was used to assess condom use when having sex in exchange for money or gifts. The participants answered these questions on a Likert scale with six-point response options ranging from (1) “always” to (6) “never.” Those answering “always” to the questions were considered consistent condom users.

The respondents were also questioned whether they had clients who requested them not to use condoms in the past 3 months (0 = no, 1 = yes), whether they were able to find condoms when they needed in the past 3 months (0 = no, 1 = yes), whether they had been diagnosed with an STI in the past 3 months (0 = no, 1 = yes), and whether they had been tested for HIV in the past 6 months (0 = no, 1 = yes). History of induced abortion was assessed via a question, “During your work as a FEW, have you experienced any induced abortion?” with three response options: (1) never had sexual intercourse, (2) no, or (3) yes. In addition, a yes/no question was used to ask whether the participants were currently using any contraceptive method.

Adverse childhood experiences (ACEs) were measured using five questions adapted from the brief screening version of the Childhood Trauma Questionnaire [26, 27]. The questions collected information on the experience of physical abuse, emotional abuse, sexual abuse, physical neglect, and emotional neglect with five response options ranging from (1) ‘never’ to (5) ‘very often.’ Participants who responded ‘never’ and ‘rarely’ were grouped together as those without ACEs, and those who answered ‘sometimes,’ ‘often,’ and ‘very often’ as those with ACEs. The Cronbach’s alpha for the Childhood Trauma Questionnaire among FEWs in this study was 0.68.

We also adapted five items from the brief screening version of the Childhood Trauma Questionnaire to enquire about family dysfunction [26, 27]. The items collected information on ‘witnessing violence against a family member,’ ‘having an alcoholic or drug user family member,’ ‘having a family member who was depressed, mentally ill, or who has attempted suicide,’ ‘having parents who had been separated or divorced,’ and ‘having a family member who has been to prison.’ The response options for all the items were ‘yes’ or ‘no,’ except for ‘having parents who had been separated or divorced.’ For this item, another response option was added to indicate if one or both parents had died. In the analysis, participants whose parents had divorced or separated were grouped together with participants whose parent (s) had died.

A short version of the General Health Questionnaire (GHQ-12) [28] was used to measure psychological distress. GHQ-12 has been validated in Asian populations [29, 30]. Each item was rated on a four-point Likert’s scale ranging from “0 = less than usual” to “3 = much more than usual.” The scoring method ‘0-0-1-1’ was adapted meaning that responses of 0 or 1 were coded “0” and responses of 2 or 3 were coded “1.” This has been suggested as it is believed to help eliminate biases resulted from respondents who tend to choose responses 0 and 3 or 1 and 2 [31]. The mean score for the whole sample was used as the cut-off to define lower and higher levels of psychological distress as it provides a rough guide to the best threshold [32]. The Cronbach’s alpha for the GHQ-12 among FEWs in this study was 0.76.

### Data analyses

Double data entry was performed using EpiData version 3 (Odense, Denmark). In bivariate analyses, we used *χ* ^2^ test, or Fisher’s exact test when sample sizes were smaller than five in one cell, for categorical variables, and Student’s *t*-test for continuous variables to compare socio-demographic characteristics, self-rated overall health and quality of life, self-perception of HIV risk, substance use, and sexual behaviors among FEWs who had a lower level of psychological distress (GHQ-12 ≤ 3) to those among FEWs who had a higher level of psychological distress (GHQ-12 > 3).

A multivariate logistic regression model was then constructed. First, we included all variables associated with psychological distress in bivariate analyses at a level of *p* <0.05 in the model. All variables with a *p*-value >0.05 were then removed from the model, and the model was refitted. The steps were repeated until *p*-values of all remaining variables were <0.05 in the final model. Adjusted odds ratio (AOR) were obtained and presented with 95 % confidence intervals (CI) and *p*-values. SPSS version 22 (IBM Corporation, New York, USA) was used for all statistical analyses.

### Ethical statement

The National Ethics Committee for Health Research of the Ministry of Health, Cambodia approved this study (Reference no. 082NECHR). A written informed consent was obtained from each participant after they were made clear that participation in this study was voluntary, and that they could refuse or discontinue their participation at any time. We protected privacy of the respondents by conducting the interviews at a private place, and no personal identifiers were collected in the questionnaires or field notes.

## Results

This study included 657 FEWs, 78.5 % from Phnom Penh and 21.5 % from Siem Reap. Of this total, 43.2 % had GHQ-12 scores of greater than 3, indicating that they have high levels of psychological distress. Descriptive statistics of demographic characteristics are presented in Table 1. The mean age was 25.6 years old (SD = 5.5), and the mean years of formal education completed was 6.4 (SD = 3.1). Marital status included never married (44.1 %), married and living together (28.6 %), and divorced/separated/widowed (27.2 %). Women worked at Karaoke bars (44.4 %), restaurants (30.6 %), and other establishments (25.0 %) including beer gardens, nightclubs, bars, massage parlors, and streets. Their average monthly income was $220 (SD = 207). On average, women worked in entertainment careers for 28.2 months (SD = 32.6) and worked at their current venue for 18.0 months (SD = 25.0). A significantly higher proportion of women with a higher level of psychological distress reported having never been married (GHQ-12 ≤ 3 = 40.8 % vs. GHQ-12 < 3 = 48.6 %, *p* = 0.04).

**Table 1:** Comparisons of characteristics of FEWs with a lower and higher level of psychological distress

Socio-economic characteristics	Total	Total GHQ-12 score		
	(*n* = 657)	≤3 (*n* = 373)	>3 (*n* = 284)	*p*-value^*^
Provinces				0.25
Phnom Penh	516 (78.5)	287 (76.9)	229 (80.6)	
Siem Reap	141 (21.5)	86 (23.1)	55 (19.4)	
Mean age (in year)	25.6 ± 5.5	25.8 ± 5.4	25.4 ± 5.5	0.40
Years of formal education completed	6.4 ± 3.1	6.1 ± 3.3	6.6 ± 3.0	0.06
Marital status				0.04
Never married	290 (44.1)	152 (40.8)	138 (48.6)	
Married and living together	188 (28.6)	121 (32.4)	67 (23.6)	
Divorced/separated/widowed	179 (27.2)	100 (26.8)	79 (27.8)	
Currently place of employment				0.84
Karaoke bar	292 (44.4)	169 (45.3)	123 (43.3)	
Restaurant	201 (30.6)	111 (29.8)	90 (31.7)	
Other^a^	164 (25.0)	93 (24.9)	71 (25.0)	
Average monthly income (in USD)	220 ± 207	222 ± 245	218 ± 147	0.82
Working duration in this career^b^	28.2 ± 32.6	30.2 ± 34.6	25.9 ± 30.6	0.10
Working duration for current venue^b^	18.0 ± 25.0	19.5 ± 25.4	16.4 ± 24.6	0.12
Tested for HIV in the past 6 months	347 (52.8)	206 (55.2)	141 (49.6)	0.16
Self-perception of HIV risk compared to the general population				0.002
Higher	148 (22.5)	64 (17.2)	84 (29.6)	
Same	92 (14.0)	56 (15.0)	36 (12.7)	
Lower	337 (51.3)	207 (55.5)	130 (45.8)	
Don’t know	80 (12.2)	46 (12.3)	34 (12.0)	
Self-rated overall health				<0.001
Good	533 (81.1)	328 (87.9)	205 (72.2)	
Poor	124 (18.9)	45 (12.1)	79 (27.8)	
Self-rated quality of life				<0.001
Good	547 (83.3)	339 (90.9)	208 (73.2)	
Poor	110 (16.7)	34 (9.1)	76 (26.8)	
Ever thought to commit suicide	128 (19.5)	45 (12.1)	83 (29.2)	<0.001
Ever attempted to commit suicide	48 (7.3)	17 (4.6)	31 (10.9)	0.08

*Abbreviations*: *FEWs* female entertainment workers, *GHQ* general health questionnaire

Values are number (%) for categorical variables and mean ± SD for continuous variables

^*^Chi-square test was used for categorical variables and Student’s *t*-test was used for continuous variables

^a^Other included beer gardens, night clubs, bars, massage parlors, streets, etc

^b^In months

Women reported that they felt they were at higher (22.5 %), the same (14.0 %), and lower (51.3 %) HIV risk compared to the general population. Most women reported good overall health (81.1 %) and good quality of life (83.3 %). Of the total, 19.5 % reported ever considering suicide, and 7.3 % reported ever attempting to commit suicide. A significantly higher proportion of women with a higher level of psychological distress reported a perception that their HIV risk was higher compared to the general population (17.2 % vs. 29.6 %, *p* = 0.002), that their overall health was “poor” (12.1 % vs. 27.8 %, *p* < 0.001), that their overall quality of life was “poor” (9.1 % vs. 26.8 %, *p* < 0.001), and that they had thought about committing suicide in the past 3 months (12.1 % vs. 29.2 %, *p* < 0.001).

Substance use among the participants is presented in Table 2. In the past 3 months, women reported having had at least one full glass of alcohol (92.7 %) and being forced to drink at work (28.0 %). In the last month, women reported getting drunk an average of 18.7 days (SD = 12.6) and consuming an average of 6.2 cans or glasses of alcohol per day (SD = 7.7). In the past 3 months, 1.7 % of women reported using any kind of illicit drugs. A significantly higher proportion of women with a higher level of psychological distress reported having had a full glass or a can of alcohol (90.9 % vs. 95.1 %, *p* = 0.04), having been forced to drink at work (19.8 % vs. 38.3 %, *p* > 0.001), and having used illicit drugs (0.5 % vs. 3.2 %, *p* = 0.01) in the past 3 months.

**Table 2:** Comparisons of substance use among FEWs with a lower and higher level of psychological distress

Substance use in the past 3 months	Total	Total GHQ-12 score		
	(*n* = 657)	≤3 (*n* = 373)	>3 (*n* = 284)	*p*-value^*^
Drunk at least a full glass of alcohol	609 (92.7)	339 (90.9)	270 (95.1)	0.04
Mean number of days getting drunk (past month)	18.7 ± 12.6	18.4 ± 12.6	19.2 ± 12.5	0.46
Mean amount of alcohol per day (cans, glasses)	6.2 ± 7.7	5.7 ± 7.3	6.7 ± 8.2	0.11
Had been forced to drink at work in past 3 months	170 (28.0)	67 (19.8)	103 (38.3)	<0.001
Used any kinds of illicit drugs	11 (1.7)	2 (0.5)	9 (3.2)	0.01

*Abbreviations*: *FEWs* female entertainment workers, *GHQ* general health questionnaire

Values are number (%) for categorical variables and mean ± SD for continuous variables

^*^Chi-square test was used for categorical variables and Student’s *t*-test was used for continuous variables

Sexual and reproductive health behaviors are reported in Table 3. Women reported a mean number of 2.8 sexual partners (SD = 6.5) in the past 3 months. Of the total, 37.2 % of women reported having sweethearts or romantic relationships and of those, 30.7 % reported always using condoms with their sweethearts. Of the total, 22.8 % reported having had sex in exchange for money in the past 3 months and of those, the mean number of clients was 3.6 (SD = 3.9). Women reported being able to find condoms when needed (87.5 %), always using condoms with clients (78.7 %), and having had clients requesting not to use condoms (16.7 %) in the past 3 months.

**Table 3:** Comparisons of sexual and reproductive health among FEWs with a lower and higher level of psychological distress

*SRH characteristics in the past 3 months*	Total	Total GHQ-12 score		
	(*n* = 657)	≤3 (*n* = 373)	>3 (*n* = 284)	*p*-value^*^
Mean number of sex partners	2.8 ± 6.5	2.6 ± 6.6	3.2 ± 6.7	0.26
Had sex with sweethearts	203 (37.2)	95 (31.7)	108 (43.9)	0.003
Always used condoms with sweethearts	63 (30.7)	33 (34.7)	29 (27.1)	0.24
Had sex in exchange for money or gifts	124 (22.8)	61 (20.4)	63 (25.7)	0.14
Mean number of clients	3.6 ± 3.9	3.2 ± 2.8	4.0 ± 4.8	0.28
Able to find condom when needed	421 (87.5)	246 (91.8)	175 (82.2)	0.004
Always used condom with clients	100 (78.7)	51 (81.0)	49 (76.6)	0.16
Had client request not to use condoms	21 (16.7)	5 (7.9)	16 (25.4)	0.009
Diagnosed with an STI	149 (22.7)	64 (17.2)	85 (29.9)	<0.001
Currently using a contraceptive method	249 (37.9)	127 (34.0)	122 (43.0)	0.02
Been tested for HIV in the past 6 months	347 (52.8)	206 (55.2)	141 (49.6)	0.16
Had been pregnant	401 (61.0)	229 (61.4)	172 (60.6)	0.83
Had an induced abortion	118 (18.0)	60 (16.1)	58 (24.0)	0.15

*Abbreviations*: *FEWs* female entertainment workers, *GHQ* general health questionnaire, *SRH* sexual and reproductive health, *STIs* sexually transmitted infections

Values are number (%) for categorical variables and mean ± SD for continuous variables

^*^Chi-square test or Fisher’s exact test was used as appropriate for categorical variables and Student’s *t*-test was used for continuous variables

In the past 3 months, 22.7 % of women reported being diagnosed with an STI, and 37.9 % reported currently using a contraceptive method. In the past six months, 52.8 % had been tested for HIV. Of the total, 61.0 % reported having ever been pregnant, and 18.0 % reported having ever had an induced abortion.

A significantly higher proportion of women with a higher level of psychological distress reported having had sex with a sweetheart (31.7 % vs. 43.9 %, *p* = 0.003), but a lower proportion of them reported being able to find condoms when they needed (91.8 % vs. 82.2 %, *p* = 0.004). Moreover, a significantly higher proportion of women with a higher level of psychological distress reported having had a client requesting not to use condoms (7.9 % vs. 25.4 %, *p* = 0.009), having been diagnosed with an STI (17.2 vs. 29.9 %, *p* < 0.001), and using a contraceptive method (34.0 % vs. 43.0 %, *p* = 0.02).

Table 4 presented ACEs and family dysfunction among FEWs in this study. During childhood, women described being physically hurt that needed medical care (26.0 %), having a family member who said hurtful or insulting things to them (25.3 %), having had someone who touched them in a sexual way (19.6 %), having had someone who took care and protected them (85.5 %), and having had someone in their family who made them feel loved (90.3 %). Within their families, women reported having a parent or guardian who had been physically abused (28.0 %), having a family member with a drinking or drug use problem (37.0 %), had a family member that was depressed or mentally ill (24.5 %), having had parents that were ever separated or divorced (18.9 %), and having a family member who had ever been to prison (7.2 %).

**Table 4:** Comparisons of adverse childhood experiences and family dysfunction among FEWs with a lower and higher level of psychological distress

Adverse childhood experiences and family dysfunction	Total	Total GHQ-12 score		
	(*n* = 657)	≤3 (*n* = 373)	>3 (*n* = 284)	*p*-value^*^
*Adverse childhood experiences*				
Physically hurt that needed medical care	171 (26.0)	72 (19.3)	99 (34.9)	<0.001
Family member said hurtful or insulting things to	166 (25.3)	67 (18.0)	99 (34.9)	<0.001
Someone touched in a sexual way	129 (19.6)	58 (15.5)	71 (25.0)	0.003
Had someone to take care of or protect	562 (85.5)	332 (89.0)	230 (81.0)	0.004
Someone in family made me feel that I was loved	593 (90.3)	341 (91.4)	252 (88.7)	0.25
*Family dysfunction*				
Parent or guardian had been physically abused	184 (28.0)	75 (20.1)	109 (38.4)	<0.001
Family member was a problem drinker/drug user	243 (37.0)	115 (30.8)	128 (45.2)	<0.001
Family member had been depressed/mentally ill	161 (24.5)	64 (17.2)	97 (34.2)	<0.001
Parents ever been separated or divorced	124 (18.9)	65 (17.4)	59 (20.8)	0.39
Family member had been to prison	47 (7.2)	18 (4.8)	29 (10.2)	0.008

*Abbreviations*: *FEWs* female entertainment workers, *GHQ* general health questionnaire

Values are number (%)

^*^Chi-square test was used

Of these experiences, a significantly higher proportion of women with a higher level of psychological distress reported having been physically hurt (19.3 vs. 34.9 %, *p* < 0.001), having had family members say hurtful or insulting things (18.0 % vs. 34.9 %, *p* < 0.001), and having been touched in a sexual way (15.5 % vs. 25.5 %, *p* = 0.003). Furthermore, a significantly lower proportion of them reported having had someone to take care of or protect them when they were growing (89.0 % vs. 81.0 %, *p* = 0.004). Regarding family dysfunction, a significantly higher proportion of women with a higher level of psychological distress reported having had a parent who was physically abused (20.1 % vs. 38.4 %, *p* < 0.001), having had a family member with a drinking or drug problem (30.8 % vs. 45.2 %, *p* < 0.001), having had a family member who was depressed or mentally ill (17.2 % vs. 34.2 %, *p* < 0.001), and having had a family member who had been to prison (4.8 % vs. 10.2 %, *p* = 0.008).

The results of multivariate logistic regression analysis are presented in Table 5. Controlling for confounding factors, women with a higher level of psychological distress remained significantly more likely to rate their overall health as poor (AOR = 1.88, 95 % CI 1.20 to 2.94), rate their quality of life as poor (AOR = 2.39, 95 % CI 1.47 to 3.87), have suicidal ideation in the past three months (AOR = 2.41, 95 % CI 1.45–3.76), rate their HIV risk as higher than the general population (AOR = 0.48, 95 % CI 0.31 to 0.74), have been forced to drink at work in the past three months (AOR = 1.77, 95 % CI 1.19 to 2.62), have had clients requesting not to use a condom (AOR = 3.48, 95 % CI 1.14 to 10.62), be not able to find condoms when they needed (AOR = 0.64, 95 % CI 0.45 to 0.93), have a family member who said hurtful things to them during childhood (AOR = 1.84, 95 % CI 1.24 to 2.75), and have a parent or guardian who had been physically abused (AOR = 1.93, 95 % CI 1.34 to 2.82).

**Table 5:** Factors associated with levels of psychological distress among FEWs in multiple logistic regression model

Variables	Total score of GHQ-12	
	AOR (95 % CI)	*p*-value
Self-rated overall health		
Good	Reference	
Not good	1.88 (1.20–2.94)	0.006
Self-rated quality of life		
Good	Reference	
Not good	2.39 (1.47–3.87)	<0.001
Having suicide ideation in the past 3 months		
No	Reference	
Yes	2.41 (1.54–3.76)	<0.001
Self-perception of HIV risk compared to the general population		
Higher	Reference	
Same	0.47 (0.26–0.84)	0.01
Lower	0.48 (0.31–0.74)	0.001
Don’t know	0.45 (0.24–0.84)	0.01
Had been forced to drink at work in the past 3 months		
No	Reference	
Yes	1.77 (1.19–2.62)	0.005
Had clients requested not to use condoms		
No	Reference	
Yes	3.48 (1.14–10.62)	0.03
Able to find condoms when they needed it		
No	Reference	
Yes	0.64 (0.45–0.93)	0.02
Family member said hurtful or insulting things to		
No	Reference	
Yes	1.84 (1.24–2.75)	0.003
Parent or guardian had been physically abused		
No	Reference	
Yes	1.93 (1.34–2.82)	0.001

*Abbreviations*: *AOR* adjusted odds ratio, *CI* confidence interval, *FEWs* female entertainment workers, *GHQ* general health questionnaire

## Discussion

Almost half of the FEWs in our study experienced high levels of psychological distress (43.2 %). It is difficult to compare this proportion with other studies because most studies focus on mental health of FSWs, yet not all FEWs are engaging in transactional sex, and because mental health is measured differently in each study. In China, two studies measured depression by using the Center for Epidemiologic Studies Depression Scale and found that 62 % [9] and 49 % [33] of FSWs had a high level of depressive symptoms. In Australia, a study that used Beck Depression Inventory II found that 54 % of FSWs reported experiencing severe depressive symptoms [6] and in Switzerland, a study that used the Composite International Diagnostic Interview (DIA-X) tool found that 50 % of FSWs had a psychiatric disorder [5].

Our findings suggest that poor mental health is associated with negative childhood events such as having a history of emotional abuse or having a parent or guardian who was physically abused. Several studies support the relationships that FSWs are more likely to have experienced traumatic events in childhood, and those who have are more likely to experience depression [34].

The majority of respondents in our study rated their own health and quality of life as good. This finding is not consistent with a study of FSWs in China where only 7.5 % women were satisfied or very satisfied with their life [35]. But FEWs who had poor mental health in our study were more likely to rate their overall health and quality of life as “poor.” This finding is consistent with psychological literature relating poor self-rated health to depressive symptoms [36].

Women who had more psychological distress were more likely to perceive their risk of contracting HIV as higher than the general population. This represents an accurate view of their risk since FSWs who are experiencing psychological distress are more likely to engage in HIV risky behaviors [9–13]. Still, over half of the respondents perceived the level of their HIV risk to be lower than the general population, which is an inaccurate identification of their level of risk and therefore, may be appropriate recipients of HIV education and risk awareness. Risk perception has been theorized to be an important motivation for adopting protective behaviors [37].

In our study, one in five of the women reported ever thinking about committing suicide and almost one in 10 ever attempted. This finding is similar to a study of FSWs in China that found that the prevalence of suicidal thoughts and suicide attempts in the last 6 months to be 14.3 and 8.4 % [35]. Women who had more physiological distress were more likely to report suicidal ideation in the past three months. This finding has important implications for future service delivery. Given that levels of suicidal ideation are so high, the GHQ-12 should be used as a screening tool to detect of psychological distress and prompt providers to enquire about suicidal ideation and appropriate follow-up.

Women in our study with a higher level psychological distress were more likely to report being forced to drink at work in the past three months. This is not an uncommon experience for FEWs and can be seen as an indicator of the kind of workplace environment within which they work [14, 15]. The type of work environment for FEWs can vary and depends particularly on whether a manager or supervisor pushes women to drink or protects them from that pressure. Occupational health literature provides support for the findings that employees who perceive low autonomy over their work are more likely to develop psychological disorders [38]. In October 2014, the Cambodian Ministry of Labor and Vocational Training has issued a new regulation that calls for the protection of the occupational safety, health, and labor rights of all FEWs [39]. While this regulation has not yet been fully implemented or enforced, future programming for FEWs can use this legislation to promote better work environments.

Finally, our study found that women with a higher level of psychological distress were more likely to have clients who requested not to use condoms and less likely to find condoms when they needed it. Our study design does not allow us to see which factor precedes the others, but it is likely that many of the factors previously discussed are involved in this relationship. In particular, having clients requesting not to use condoms and not being able to find condoms may be further indicators of not having control over their work environment. Condom negotiation skills and availability are two critical service needs for FEWs and should be top priorities for future programming.

Limitations of this study include the following. First, causal inferences were not possible due to the cross-sectional study design. Second, all measures were self-reported which means that there may be biases that may lead to both underreporting and over-reporting of certain variables. Cultural norms around sexuality and sexual practices in Cambodia suggest that information about sexual behaviors may be under-reported. Efforts were made to reduce these biases including study procedures that ensured confidentiality including de-identifiable information and interviews conducted in confidential locations. Third, the sample may not be representative of all FEWs in Cambodia. Data were collected only from FEWs in the capital city and a large province where the SAHACOM, a comprehensive community-based project aiming to improve sexual reproductive health of FEWs, has been implemented by KHANA. Therefore, participants had an existing link to services. The levels of sexual and reproductive health risks and outcomes reported in this study may therefore represent a more optimistic view than in other areas of Cambodia.

## Conclusions

In conclusions, FEWs in this study experience high levels of psychological distress, which likely stems from both past negative experiences and current working conditions. Women with higher psychological distress are at higher risk for poor health outcomes, including attempted suicide, substance use, and engagement in risky sexual behaviors. For women that are experiencing psychological distress, programming should specifically address substance use, condom negotiation and availability, and suicide risk.

## Abbreviations

ACEs: Adverse childhood experiences
AIDS: Acquired immune deficiency syndrome
AOR: Adjusted odds ratio
CI: Confidence interval
FEWs: Female entertainment workers
FSWs: Female sex workers
GHQ: General Health Questionnaire
HIV: Human immunodeficiency virus
SAHACOM: Sustainable Action against HIV and AIDS in Communities
SD: Standard Deviation
STIs: Sexually transmitted infections
PTSD: Posttraumatic stress disorder
